# Editorial

**Published:** 2015

**Authors:** Mircea Filip

**Affiliations:** * President of the Romanian Society of Ophthalmology

“Romanian Journal of Ophthalmology” has represented a subject of debate for a long time among the ophthalmologists; questions regarding what role it should have, how much are we interested in publishing in it, how much help we will get from it, have been our main concerns. We should not forget that the journal has appeared many years ago, when information sources were limited – its appearance was well received due to the information provided. The appearance of “Mr. Google” generated, as in many others domains, changes of the community’s attitude towards it. Many critical opinions have emerged because the journal was not able to support those who were in need of promotion, due to the lack of quotations. The editorial staff made admirable efforts, but if we make an effort to publish an article, we should get the right amount of support for it. Obtaining a quotation for the journal is a long and difficult process that requires access to a “club” of periodical publications and involves certain rules for publishing.

To summarize, there are some mandatory conditions:

• international editorial board

• representative graphic design

• respecting the date of the appearance – quarterly

• articles written only in English.

Perhaps many of us do not know that, in order to publish in a journal with international recognition, one has to pay an important amount of money - hundreds of euros or dollars. But, more than that, the journal reflects the national scientific level of our specialty and, in order to be appreciated as closer to reality as possible, it must be read by colleagues from other countries, not only by us, the Romanians. We advocate to publish the journal in an international language. The Board of the Society chose the English language. Knowing the results of the community work, being aware of its value is difficult, requires continuity, consistency and time. The journal is a reflection of what we do and where we stand, with good and bad things. This is what the Board of the Society is trying to do now, by assuming these principles and by taking this decision. I think we made a wise decision that will pay back on the long run. It is not in our advantage to continue in the same manner and the result can be seen.

Marketing is an important process that should be known everywhere. This means an effort made mainly by specialists, not only by us, in marketing. We also have to send our review to different places (libraries, universities, etc.) and to different personalities. This is something to be evaluated in time, depending on the evolution of our success with the “Romanian Journal of Ophthalmology”.

